# Intervertebral disc degeneration—Current therapeutic options and challenges

**DOI:** 10.3389/fpubh.2023.1156749

**Published:** 2023-07-06

**Authors:** Ankita Samanta, Thomas Lufkin, Petra Kraus

**Affiliations:** Department of Biology, Clarkson University, Potsdam, NY, United States

**Keywords:** intervertebral disc degeneration, therapy, exosome, small molecule, stem cell

## Abstract

Degeneration of the intervertebral disc (IVD) is a normal part of aging. Due to the spine's declining function and the development of pain, it may affect one's physical health, mental health, and socioeconomic status. Most of the intervertebral disc degeneration (IVDD) therapies today focus on the symptoms of low back pain rather than the underlying etiology or mechanical function of the disc. The deteriorated disc is typically not restored by conservative or surgical therapies that largely focus on correcting symptoms and structural abnormalities. To enhance the clinical outcome and the quality of life of a patient, several therapeutic modalities have been created. In this review, we discuss genetic and environmental causes of IVDD and describe promising modern endogenous and exogenous therapeutic approaches including their applicability and relevance to the degeneration process.

## Introduction

**Figure 1:**
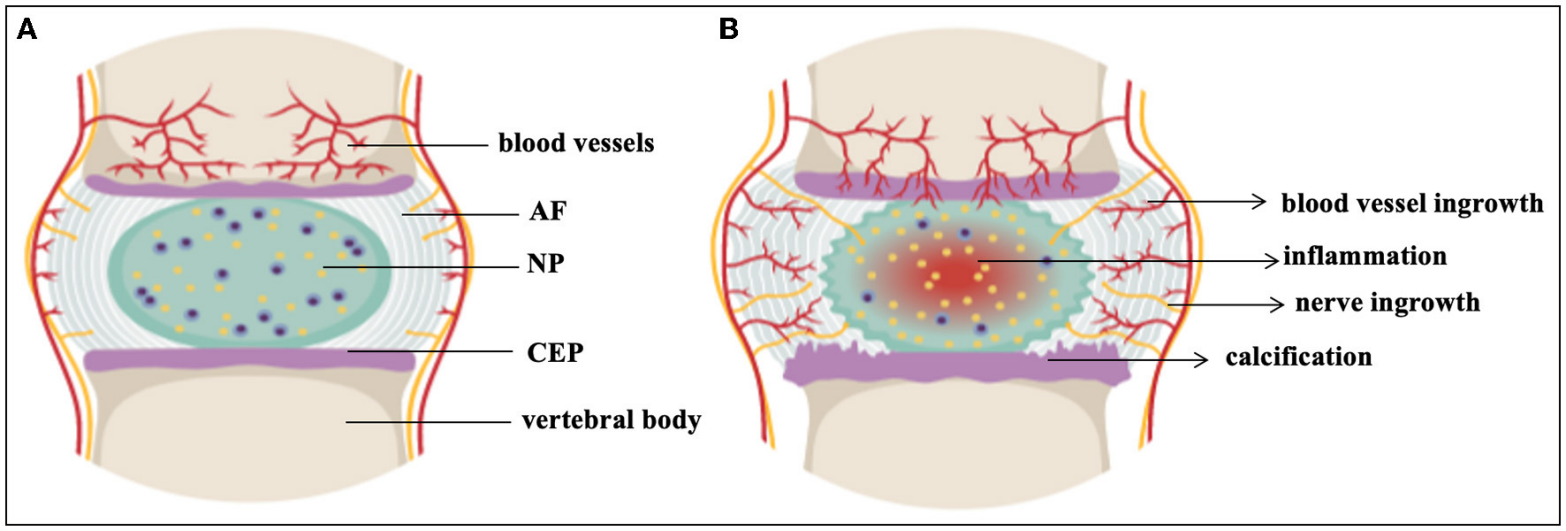
The intervertebral disc. **(A)** Healthy IVD **(B)** Degenerated IVD. AF, annulus fibrosus; CEP, cartilaginous end plates; NP, nucleus pulposus. This figure created in the Mind the Graph platform (www.mindthegraph.com).

Low back pain (LBP) is one of the most common health concerns in the world. It affects a significant part of the population and, in the United States, has the highest health-related economic cost of up to 560–630 billion dollars per year (1–3). It is estimated that between 70 and 85% of the population will experience LBP at some point in their lives and that it can already limit activities in those under the age of 45, posing a significant socioeconomic impact by accounting for over 100 million lost workdays annually in the USA alone (4, 5). LBP presents as one of the most frequent causes of disability among young adults (6, 7). Although the reasons for most cases of LBP are unknown, intervertebral disc degeneration (IVDD) is regarded as the most common factor (1, 8). IVDD is not limited to humans (9). The organ affected in IVDD is the intervertebral disc (IVD). The IVD is a semi-movable joint and a cushion of fibrocartilage between the vertebrae. It is comprised of a central nucleus pulposus (NP) surrounded by an inner and outer annulus fibrosus (AF) and is sandwiched between the cartilaginous endplates (CEP) as seen in Figure 1A (10–15). The AF is made up of concentric lamellae, which are densely interwoven collagen bundles that run obliquely between adjacent vertebral bodies (14, 16). The NP, on the other hand, has a loose collagen network and is highly hydrated (11, 14, 16). Compared to other tissue types, NP cells are present at a low cell-density (3,000 cells/mm^3^) sequestered in an abundance of extracellular matrix (ECM) while the cell density in the AF is ~3 × higher (17–19). The strong, fibrous collagen framework of the disc holds cells and proteoglycans (PG) in the matrix in place while securing the disc to the vertebral bodies (20, 21). Collagen II represents ~20% of the NP dry weight, while PG, especially the big aggregating PG aggrecan (ACAN), make up ~50% of the NP dry weight (22), the latter providing the osmotic swelling pressure that maintains disc height and turgor amidst heavy compressive loads or impacts.

During embryogenesis carefully orchestrated events give rise to the notochord (NC) a crucial structure during early chordate development that is filled with relatively large, vacuolized NC cells. NC cells take on an important function in maintaining hydro-pressure against external forces through the production of polar macromolecules (23, 24). While largely considered conserved, precise events of early notochord generation might vary between different chordate species (25). Genetic engineering in mouse demonstrated not only that the NP is of NC origin but also identified the expression of many important transcription and signaling factors that are involved in these early patterning events such as *Shh, Sox5*, Sox*6* and Sox*9*; the Paired box genes *Pax1* and *Pax9* alongside *Nkx3.2* (*Bapx1*), *Noto* and *Brachyury* (*Tbx1*) to name a few (26–37). NC cells make up the NP of early vertebrate IVDs (38, 39) but depending on the species, these cells might be reduced to a minimum population with different ratios compared to other IVD cells, rendering the NP a heterogeneous cell population (40–44). This loss of NC cells is often correlated with the onset of disc degeneration (45). Morphology and cell composition of the adult NP can differ between mammalian species (46, 47). In some rodents popular in research NC-like cells are retained into adulthood, whereas in other animals, chondrocyte-like (CL) cells become more prominent over time. The origin of these CL cells is of debate. Transdifferentiation of NC cells into CL cells, or progenitor cell recruitment into the NP by NC cells prior to undergoing regulated cell death are considered. Previously receptor tyrosine kinase (Tek or Tie2) and disialoganglioside 2 (Gd2) expression was used to identify multipotent stem cell populations in the IVD, while the siaologlycoprotein encoding gene *CD24* plays a role in the differentiation of different cell types. *Tie2*+*/Gd2–/CD24–* progenitor cells were identified in the mouse, human and bovine NP and described as dormant stem cells (47–49). A gradual change from a *Tie2*+*/Gd2*+*/CD24*– phenotype with self-renewal potential and stem cell properties to a *Tie2*–*/Gd2*+*/CD24*– phenotype of potential and a *Tie2*–*/Gd2*+*/CD24*+ phenotype of committed NP progenitors before committing to a mature NP phenotype (*Tie2*–*/Gd2*–*/CD24*+) was suggested (47, 48). Recent single cell RNA sequencing (scRNASeq) analysis of sorted murine NP cells from 1 month old C57BL/6 mice identified four subpopulations. One with an enrichment of stemness genes was considered as NP progenitor cells involved in the regulation of cell growth and differentiation based on their transcription factor profile (50). A mouse NP progenitor cell population expressing the G-protein-coupled receptor *Uts2R* was located in the peripheral NP, with the majority of these cells expressing *Tie2* and ~1/3 *Tie2* and *Gd2*. The authors demonstrated that this progenitor population declines in IVDD (50) supporting work pioneered by Sakai et al. (47). It was further suggested through *Krt19* fate mapping that CL cells in the lumbar NP of mice older than 18 months represent a NP cell derived terminal differentiation stage and that < 15% thereof remain *Shh* positive, suggesting age-related transdifferentiation over cell invasion (51). In the human NP, NC cells disappear in early childhood (4–10 years of age) (46, 52, 53). ScRNASeq recently deciphered several cell clusters in the healthy human IVD, amongst them three chondrocyte subclusters with many cells expressing *Noggin* (*NOG)*, a small group of cells expressing NC markers *TBXT* and *KRT8* and a group of multipotent NP progenitor cells expressing *PROCR*, a gene associated with signaling receptor activity and stemness, and *PDGFRA* associated with mesenchymal stem cells (MSC), molecular evidence suggesting that different cell morphologies in the NP reflect phases of NC lineage cells during aging and degeneration (54). Based on these findings a correlation between declining numbers of *UTS2R*+ or TIE2+/GD2+ stem-like cells and the onset of IVDD would also be expected, yet was not specifically described for human NP CL cell populations of various IVDD degrees (55, 56), however the relevant stages might have been missed.

One of the earliest changes in IVDD is a loss of PG content and composition, resulting in reduced hydration, height and flexibility of the disc (11) as seen in Figure 1B. IVDD is a chronic disorder characterized by a progressive loss of mechanical stability and shock absorber function, which can lead to the formation of osteophytes and restricted motion in spinal segments (57). IVDD is frequently associated with spondylolisthesis, disc herniation, sciatica, spinal canal stenosis, and degenerative scoliosis (58). About 20% of teenagers show signs of beginning IVDD (5, 59) including athletes, especially those involved in high impact sports such as football, gymnastics and diving. While frequencies of cervical spine injuries were studied amongst school-age and college athletes (60, 61), IVD damage amongst this cohort is less well documented (62). IVDD also affects the ability of the spine to resist physiologically acceptable loads during daily activities, and impacts on the function of adjacent tissues, such as the muscles and ligaments (63). Chronic LBP continues to limit abilities and the quality of life for a large percentage of the population, despite access to invasive and expensive surgical interventions for discogenic pain such as arthroplasty and arthrodesis (5, 64). Restoring the ECM components of the IVD to their initial state would be preferable (65). Therefore, initiatives have been undertaken to create non-operative therapy modalities that are both efficient and secure. A major area of study is the direct injection of active compounds to prevent, slow down, or even reverse IVDD (66, 67). In the past 30 years, numerous clinical trials investigating biologic, cell- and scaffold-based injectable therapies for symptomatic IVDD have been undertaken (6, 17, 68). Several preclinical animal studies and fundamental scientific investigations support each of the clinical trials (69) (Table 1). However, scientifically established methods to prevent or reverse IVDD and the accompanying discogenic pain are not yet available. The current lack of success in treatments demonstrates the complexity of this illness (137). In this review, we present the status of IVDD causes alongside the challenges of available therapies.

**Table 1:** Preclinical studies for different interventions using various model systems to assess therapeutic potentials in IVDD.

**Preclinical** ***in vivo*** **studies on growth factors**			
**Animal model**	**Therapeutic source**	**Brief outcome**	**References**
Rabbit	PGDF-BB	Alleviated disc degeneration, prevented apoptosis	(70)
	OP-1	Disc height increased Disc height and proteoglycans increased	(71–73)
	BMP-2	Increase of hypervascularity and fibroblast proliferation	(74)
	GDF-5	Increased cell proliferation and matrix synthesis	(75)
	PRP	Disc height increased along with chondrocyte proliferation	(76)
	PRP	PRP-ADSC group restored discs compared to controls.	(77)
Rat	IGF-1, GDF-5, TGFβ, bFGF	Increase of GDF-5 and TGFβ	(78)
	GDF-5	Slows progression of degeneration	(79)
Mouse	GDF-5	Disc height increased	(80)
Dog	NTG-101	Decreased expression of pain related neutrophins	(81)
**Preclinical** ***in vitro*** **and** ***in vivo*** **studies on EVs**				
**EV source**	**Method**	**Animal model**	**Brief outcome**	**References**
AD-MSCs	*In vitro*	Human	NPCs were protected from oxidative stress by the lyo-secretome	(82)
BM-MSCs	*In vitro*	Mouse	Increase in Col2 and Acan expression	(83)
	*In vivo*		Decreased levels of Mmp3 and Mmp6	
	*In vitro*	Mouse	Reduced inflammatory cytokines and activated MAPK pathway	(84)
	*In vitro*	Rat	Inhibition of apoptosis and ECM catabolism	(85)
	*In vitro*	Rat	Decreased NPC apoptosis	(86)
	*In vivo*		Slowed the decrease in disc height	
	*In vitro*	Rat	Apoptosis decreased for NPCs in treatment group.	(87)
	*In vivo*		Alleviated expression of Tnf-α	
	*In vitro*	Human	Proliferation rate increased	(88)
	*In vitro*	Human	Reduction of ER stress-induced apoptosis	(89)
	*In vitro*	Human	Apoptosis reduced in degenerated disc cells.	(90)
	*In vitro*	Human	Upregulation of COL2A1 and ACAN	(91)
	*In vitro*	Human	Inhibition of AF cell autophagy	(92)
UC-MSCs	*In vitro*	Human	Prevented damage from high glucose induced injury	(93)
USCs	*In vitro*	Human	Lowered GRP78, GRP94, Caspase 3, and Caspase 12 expression	(94)
	*In vivo*	Rat	Alleviated IVDD *in vivo*	
MSCs	*In vitro*	Rat	Inhibition of apoptosis Alleviates IVDD	(95)
	*In vivo*		Alleviated IVDD hallmarks	
	*In vitro*	Mouse	Inhibited pyroptosis	(96)
	*In vivo*		Alleviated IVDD	
PLMSCs	*In vitro*	Human	Induces proliferation and migration	(97)
	*In vivo*	Mouse	Increased ZNF121 expression	
NCs	*In vitro*	Canine	Increased GAG and collagen content	(98)
		Human	Increased GAG and collagen content	
	*In vitro*	Bovine	Only canine CLCs were affected by the mild concentration-dependent anabolic impact of EVs.	(99)
		Canine		
	*In vitro*	Human	Angiogenesis was inhibited by EV conditioned media via miR-140-5p, which also controls WNT/Catenin signaling.	(100)
	*In vivo*	Mouse	Vascularization in degenerated IVDs was inhibited by EV conditioned media.	
NPCs	*In vitro*	Rat	Upregulation of *Acan, Sox9*, and *Col2a1* compared to controls	(101)
	*In vitro*	Human	Increased expression of ACAN, SOX9, COL2A1, HIF1a, CA12, and KRT19	(88)
	*In vivo*	Rat	miR-223-3p application lowered C-fiber responses	(102)
	*In vitro*	Rat	*P21* and *P53* relative expression increased in senescent NPC EVs.	(103)
	N/A	N/A	NPC autophagy and EV secretion were induced by rapamycin and bafilomycin A1 in an autophagy-dependent manner.	(104)
	*In vitro*	Human	Downregulation of SIRT1 *in vitro*	(105)
	*In vivo*	Rat	By adsorbing miRNA-141-5p and downregulating SIRT1 *in vivo*, circRNA_0000253 accelerated IVDD.	
	*In vitro*	Human	Significant cellular uptake	(106)
AFCs	*In vitro*	Human	Degenerated AFC-EVs stimulated cell migration and increased levels of IL-6, TNF-α, MMP-3, MMP-13, and VEGF, whereas EVs originating from non-degenerated AF cells had the opposite effects.	(107)
CEPCs	*In vitro*	Rat	Apoptotic bodies promoted PPi metabolism, increased Pi and decreased PPi	(108)
CESCs	*In vitro*	Rat	Alleviation of IVDD by the activation of the PI3K/AKT pathway	(109)
PMEFs	*In vivo*	Mouse	Upregulation of Foxf1 and Brachyury	(106)
**Preclinical** ***in vivo*** **and** ***in vitro*** **studies on gene therapy**			
**Therapeutic source**	**Animal model**	**Brief outcome**	**References**
Naringin	Rat	Might have a protective effect on IVD.	(110)
Cannabidiol	Rat	High dose can only alleviate IVDD	(111)
EGCG	Rat	Reduction of pain *in vivo*	(112)
UA	Rat	UA alleviated IVDD	(113)
E2	Rat	E2 can regulate autophagy of IVD and can be a therapeutic agent in postmenopausal women	(114)
	Rat	E2 downregulates catabolic proteins and prevents IVDD	(115)
Icariin	Rat	Icariin reduced disruption of AF	(116)
Resveratrol	Rabbit	Resveratrol alleviated IVDD	(117)
	Rat	Levels of IL-1 and TNF-α proteins decreased	(118)
CXB	Dog	In dogs with IVDD, the controlled dose of CXB partially inhibited the generation of PGE2.	(119)
	Dog	*In vivo*, the progression of IVDD was reduced by intradiscal regulated release of CXB. Life quality improves without evident signs of regeneration	(120)
Berberine	Rat	Could alleviate IVDD in animal model	(121)
Metformin	Rat	Showed a protective effect against IVDD	(122)
Gefitinib	Rat	Decreased histological scores in comparison to the control group	(123)
Statin	Rat	Intradiscal injection alleviates IVDD	(124)
Luteoloside	Rat	ECM and NP tissues well preserved	(125)
Curcumin	Rat	Lowered NF-κB-p65 and TNF-α expression	(126)
**Preclinical** ***in vivo*** **and** ***in vitro*** **studies on gene therapy**			
**Vector**	**Method**	**Animal model**	**Reference**
BV	*In vivo*	Rabbit	(127)
LV	*In vivo*	Rabbit	(128)
RV	*In vitro*	Bovine	(129)
AV	*In vitro, In vivo*	Rabbit	(130)
AAV	*In vivo*	Rat	(132)
RNAi	*In vitro*	Rat	(133)
Ultrasound targeted microbubble destruction	*In vivo*	Rat	(134)
Polyplex micelle	*In vitro*	Human and rat	(135)
CRISPR/Cas9	*In vitro*	Human	(136)

ACAN, Aggrecan; ADSC, adipose-derived mesenchymal stromal cell; AV, adenovirus; AAV, adeno-associated virus; AFC, annulus fibrosus cell; bFGF, basic fibroblast growth factor; BM-MSC, bone marrow-derived mesenchymal stem cell; BMP, bone morphogenetic protein; BV, baculovirus; CEPC, cartilage endplate chondrocyte; CESC, cartilage endplate stem cell; CLC, chondrocyte like cell; CXB, celecoxib; COL, collagen; E2, estradiol; ECM, extracellular matrix; ER, endoplasmic reticulum; EV, extracellular vesicle; GAG, glycosaminoglycan; GDF, growth differentiation factor; IGF, insulin like growth factor; IL, interleukin; IVDD, intervertebral disc degeneration; LV, lentivirus; MAPK, mitogen activated protein kinase; MMP, matrix metalloproteinase; MSC, mesenchymal stem cell; NC, notochordal cell; NF-kb, nuclear factor kappa-light-chain-enhancer of activated B cells; OP, osteogenic protein; Pi, inorganic phosphate; PPi, extracellular pyrophosphate; PGDF-BB, platelet-derived growth factor BB; Pi, inorganic phosphate; PI3K/AKT, phosphatidylinositol 3-kinase Akt; PLMSC, placental mesenchymal stem cell; PMEF, primary mouse embryonic fibroblast; PPi, extracellular pyrophosphate; PRP, platelet rich plasma; RNAi, RNA interference; RV, retrovirus; SIRT, Sirtuin; TGF, transforming growth factor; TNF, tumor necrosis factor; UA, urolithin A; UC-MSC, umbilical cord-derived mesenchymal stem cell; USC, urine-derived stem cell.

## Causes of IVDD

**Figure 2:**
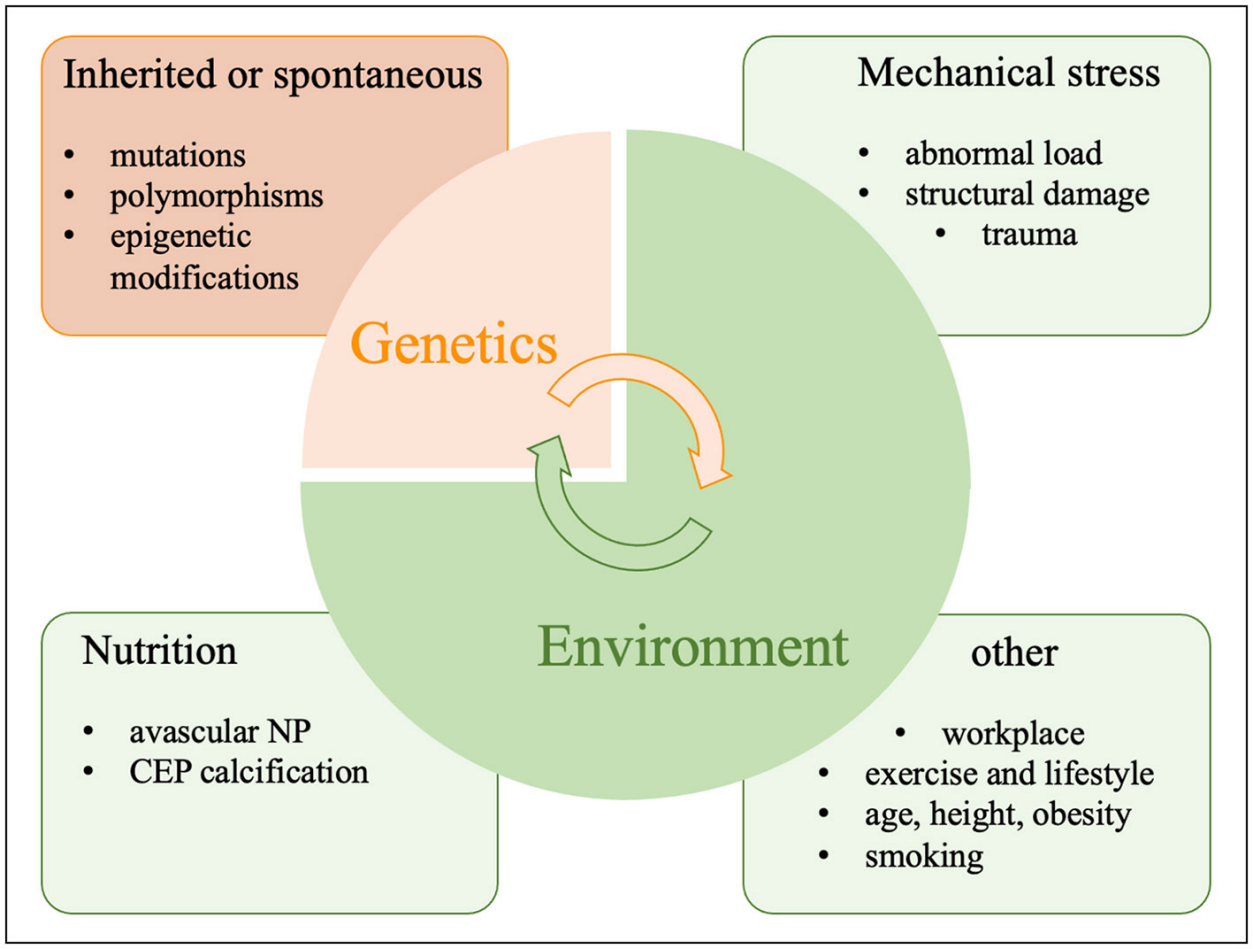
Causes of IVDD.

IVDD is usually caused by a conflux of genetic, environmental and lifestyle factors as well as trauma as seen in Figure 2. Therefore, the genetic and environmental risk factors outlined here might each or in combination contribute to and aggravate IVDD as seen for comorbidities in other chronic illnesses (138). Magnetic resonance imaging (MRI) has improved classifications of disc degeneration (139–141). IVDD frequently occurs when ECM catabolism outweighs its anabolism (142). The pathophysiology of IVDD is influenced by many other factors such as genetics and the environment including an unhealthy lifestyle, inactivity, smoking, occupational exposure to vibration, mechanical loading, severe trauma, psychosocial problems, benefit payments and more (143, 144).

### Genetic factors

IVD cells are impacted by changes in ECM composition, structure and function resulting from genetic polymorphisms and DNA mutations (Table 2) (162, 163). Recent research on heredity and linkage has undoubtedly increased awareness of genetic predisposition to IVDD. However, the degree and kind of genetic influences are still not fully understood. The association between disc degeneration and genetic polymorphisms such as variable number of tandem repeats (VNTR) or single nucleotide polymorphisms (SNP) of certain ECM macromolecules is considered a main genetic factor (164, 165). For example, polymorphisms in PG encoding genes such as *ACAN* were associated with IVDD. Polymorphisms affecting fibrillar collagen ECM constituents like COL11A1 were reported in the context of disc herniation and IVDD (166–169). Transgenic mice with a mutation in *Col9a1* demonstrated progressive IVDD, likely affecting synthesis or assembly of non-fibrillar Col9a1 chains (170); while *COL9A2* and *COL9A3* variants were significantly correlated with sciatica and lumbar disc degeneration in a Finnish population (171, 172). Other genetic associations involve ECM remodeling enzymes such as matrix metalloproteinases (MMP), more specifically MMP2 and MMP9, both a gelatinase and type IV collagenase and MMP3, a PG degrading enzyme (173–178). Other disintegrin, and metalloproteinases (ADAM), and those with thrombospondin motifs (ADAMTS) show changes in expression patterns during IVDD (173, 179–182). In this context, polymorphisms in the vitamin D receptor (VDR) across diverse ethnic backgrounds were linked to IVDD (168, 183–188). While the foundation for some genetic studies was based on a limited cohort size (189) the rapid development of next and third generation sequencing technologies allowed for genome wide association studies (GWAS) as seen beneficial in other fields, alongside more targeted, specific studies of susceptibility regions in large patient and control cohorts of different ethnic backgrounds. This genetic association suggests a pleiotropic nature of IVDD (190). For instance, transcriptional regulators *NFAT1* and *SOX9* control the expression of many genes that are both anabolic and catabolic and mediate ECM production (191, 192). *CHST3*, encodes for an enzyme that catalyzes the sulfation of chondroitin, an ECM PG (148). Amongst other susceptibility loci identified through GWAS are known players in the context or ECM production, chondrogenesis and cell survival like *BARX1, COL11A1, COLGALT2, TGFA, FGFR3, FOXA3, GDF5, SMAD3*, and *TGFA* (190). While most studies so far focused on caucasian populations, a recent GWAS focusing on a Chinese cohort identified polymorphisms near *Gasdermin-C (GSDMC)*. Interestingly, gasdermins are involved in mediating pyroptosis as a form of regulated cell death (193), however the studies phenotype/genetic variant association differed from previous findings (150), indicating the importance of such studies across different ethnic backgrounds as well as the need for a precise definition of IVDD phenotypes in such studies. Also, non-lethal polymorphisms in early IVD patterning genes will likely surface over time as underlying cause. Furthermore, both single cell and bulk transcript analysis of IVD derived cells through RNASeq and other methods will likely point to biomarkers for NP and AF cells of healthy or degenerated discs worth investigating in IVDD linkage analysis (41, 42, 194–198). Going forward it will be crucial to investigate not only polymorphisms in coding and regulatory regions such as promoters or enhancer/silencer binding sites of genes but also epigenetic modifications from methylation, acetylation and lactylation involved in metabolic reprogramming among other effects (197, 199). This could facilitate the identification of environmental variables as a factor in disc degeneration as IVDD is a multifactorial disease (162, 200, 201).

**Table 2:** Cohort studies in the field of IVDD.

**Published/peer reviewed/in-preprint cohort studies**			
**Summary**	**Outcome**	**Limitations**	**References**
**GWAS based studies**			
Investigate 5′ upstream SNP variant rs143383 in 5 population cohorts in Northern European women.	Positive association	IVDD struggles with a lack of established epidemiologic explanations, which makes it difficult to examine it methodically.	(145)
Investigate VNTR polymorphism in 132 middle aged Finnish men.	*ACAN* polymorphism has a correlation with IVDD.	Small sample size *N* = 132	(146)
GWAS to study chronic LBP.	Association of chronic LBP with genes expressed in the brain. Greater genetic contribution to chronic vs. acute pain.	No information on a detailed pain phenotype description or pain medication.	(147)
Linkage to CHST3 variants	*CHST3* linkage with IVDD.	Small sample size *N* = 4,043	(148)
First GWAS meta-analysis of IVDD with 4,600 subjects	*PARK2* gene is involved in IVDD.	Small sample size. *N* = 4,600	(149)
GWAS related transcriptome analysis of *Gasdermin-C*	Association ofrs6651255 and rs7833174 with lumbar spinal stenosis.	Selection bias and small sample size. *N* = 400	(150)
**Anatomical based studies**			
MRI of 200 IVDD patients on signs of degeneration with respect to age, sex, and other factors.	21–30-year-old 38.8% showed at least one IVDD symptom. 51–60-year-old 91.6% showed at least one IVDD symptom. No substantial difference in disc height amongst all groups.	Patient medical histories were not obtained. Area of facet joint arthritis was not included. Quantification of spondylosis was not investigated.	(151)
Investigation of LSTV with LBP and IVDD	IVDD and LBP had a correlation with LSTV.	Small sample size *N* = 1,468	(152)
Baggage handlers from the Copenhagen Airport vs. control group	LBP was more prevalent in the baggage handlers.	Degree of exposure. Misclassification and misinterpretation of outcomes.	(153)
Investigates correlation between short and long-term physical inactivity and degeneration of the thoracic and lumbar spine.	Physical inactivity over a period of 14 years had a strong connection to IVDD.	No prior MRI images to compare with current MRI images. Lack of substantial information about physical activity.	(154)
Investigation if different thresholds of IVDD lead to a correlation between disc degeneration and self-reported LBP.	IVDD was most strongly associated with LBP at thresholds at more moderate grades at ages 45 and 49, despite a tendency for disc degeneration to be more strongly associated with LBP at thresholds at more severe grades of disc signal and disc height loss at age 41.	Odds ratio calculated by logistic regression analysis are associated with anomalies.	(155)
Fluoroscopic studies to compare intervertebral angular motion sharing inequality and variability during continuous lumbar motion in chronic, non-specific LBP patients and controls.	Higher inequality motion sharing was found in patients with chronic, non-specific LBP.	Small sample size. *N* = 20	(156)
A lumbar radiograph and a questionnaire were completed by 699 individuals.	No correlation between osteophytes and LBP Disc space narrowing is associated with neuropathic pain.	Inconsistent quality of radiographs. Anteroposterior lumbar radiograph was not available.	(157)
Correlation of symptomatic and asymptomatic age related IVDD.	Severe degeneration was seen in symptomatic patients in comparison to the asymptomatic patients.	The symptomatic group had patients only from 30 to 79 years of age.	(158)
**Pain level based and other cohort studies**			
Investigates correlation between modic changes and LBP.	Significant and independent association between modic changes LBP.	Small sample size. *N* = 1,512	(159)
Investigates correlation between LBP, IVDD and mental distress.	Of the total population, 5.2% had severe and frequent LBP, and 29.0% had no LBP. Mental distress increased the correlation between LBP and IVDD.	Definition of clinically significant pain was relative. Details were not obtained on other comorbidities.	(160)
Investigatescorrelation between BMI, smoking and physical activity with IVDD in young adults.	Environmental factors play a role in IVDD in young males.	The smoking data was self-reported Imaging design was cross-sectional.	(161)

ACAN, Aggrecan; BMI, body mass index; CNS, central nervous system; CHST3, carbohydrate sulfotransferase 3 variant; GWAS, genome wide association studies; IVDD, intervertebral disc degeneration; LBP, low back pain; LSTV, lumbosacral transitional vertebrae; MRI, magnetic resonance imaging; SNP, single nucleotide polymorphism; VNTR, variable number of tandem repeats.

### Environmental factors

#### Metabolic stress factors

The disc's microenvironment is complex. The healthy adult NP is the largest avascular, organ in the vertebrate body and the distance to the closest blood vessel can be up to 8 mm (202). Residing cells rely on diffusion from capillaries penetrating the outer AF and adjacent CEPs to transport nutrients or oxygen and to remove metabolic waste products. This generates challenging circumstances for NP cell survival in this unique *in vivo* niche (203–205). In a healthy NP, the oxygen tension is 2%; in a degenerated NP, it is 1% (206). These anaerobic conditions result in lactic acid fermentation for energy production (11, 203, 207, 208), which alongside proton retention via ECM-PGs renders even the healthy NP slightly acidic (~pH 7.1). The acidity increases further in the degenerate stage with pH readings of 6.5–5.7 (209–212). It was reported that the activity of disc cells is extremely sensitive to extracellular oxygen and pH *in vitro*, that ECM production rates fall sharply at acidic pH and at low oxygen concentrations, and that cells are not able to withstand extended exposure to low pH (16, 213). Decreased nutrition supply was further considered a cause of progressive IVDD with aging potentially as an implication of increased calcification and erosion of the CEP (22, 205, 214–217). Experimentally and in human patients, it has been demonstrated that disruptions in nutrition delivery have an impact on how oxygen and lactic acid are transported in and out of the disc (206). However, it was also shown that lactate can serve as carbon source for various cell types (218, 219) and NP cells in their unique niche likely developed metabolic adaptions catering to the use of lactic acid, given that primary cells isolated from a healthy coccygeal bovine IVD preferred the absence of glucose in serum containing monolayer culture (197). Disc degeneration and back discomfort are linked to conditions that influence the blood flow to the vertebral body, such as abdominal aortic atherosclerosis, increased CEP erosion and calcification (216, 217, 220). Impairment of the CEPs also alters the NP's mechanical loading, which causes alterations in the disc's metabolism (221, 222). Endplate calcification as seen in scoliotic discs can affect nutrients and metabolite transport through the endplate and further aggravate hypoxia and an acidity (223, 224). Calcified CEP with 50% reduction in permeability resulted in disc deformity and a drop in IVD glucose levels to half of levels in the healthy NP (225, 226). This could prevent IVD cells from sustaining the ECM (222, 227). Additionally, it has been shown that deteriorated IVDs exhibit chronic inflammation (228), with increased expression of a number of pro-inflammatory cytokines (207). This includes interleukin (IL) 1, MMP10 (229), MMP12 (230), cyclooxygenase 2 (COX2) (231), IL8, tumor necrosis factor- (TNF) 22 (232), IL10 (233), IL2, IL4, and IL17 (234), among others, which may be strongly associated with discogenic pain (235).

#### Mechanical stress factors

The IVD is an important part of the vertebral column facilitating protection of the vertebrae and spinal cord during regular daily activities, exercise, and accidental trauma. Abnormal mechanical load and stress can lead to disc injury and degeneration. For many years, it was believed that injuries, which result in structural damage, are a major contributor to spinal disorders (236). These injuries eventually result in IVDD and associated symptoms like back pain. This finding has been supported by animal models (237). While exercise is generally considered a healthy activity, some forms including impact or strenuous loading (diving, gymnastics, weight lifting, and high impact contact sports) can trigger IVDD, while other forms are beneficial resulting in increased anabolic responses with increased glycosaminoglycan and hydration levels in the IVD (62). For instance, more lumbar IVD degeneration was seen in gymnasts compared to controls who weren't athletes (238), as well as in soccer players and weightlifters in comparison to shooters (186). Evidence supporting the positive effects of exercise on the IVD in humans, however, is less clear (239). Basketball, baseball, swimming, and soccer were linked to better IVD parameters over controls, while poorer NP hydration was marginally linked with a longer career and heavier training load (62). The transport of nutrients into the disc and, consequently, their concentration in the tissue, appears to be influenced by exercise (223, 224). Although the exact mechanism is unknown, it has been proposed that exercise alters the capillary bed's morphology at the disc-bone interface (223).

#### Other environmental factors

Risk factors such as age, low income, prior cervical spine surgery, type of health insurance, and medical comorbidities like cancer, diabetes, hypertension, depression, hypothyroidism, peripheral vascular disease chronic obstructive pulmonary disease (COPD), and lifestyle choices such as smoking were linked to IVDD (240–244). Numerous studies have linked tobacco use to lower back pain. Smoking is known to prevent the fusion and healing of bones and initially reduces the proliferation and activity of fibroblasts and osteoblasts and the usual inflammatory response (245–248). It subsequently interferes with neovascularization and the normal vascular supply, encouraging net bone resorption rather than net bone growth (245, 247). Notably, after lumbar or cervical fusion surgery, pseudarthrosis occurs at a rate that is two times higher among smokers (245, 249–252). Tobacco inhalation and nicotine caused vasoconstriction and decreased the exchange of nutrients and anabolic substances, resulting in inadequate IVD nourishment, ECM and NP cell development all contributing to the IVD's instability and degeneration (253–258). Toxins from cigarette smoke impaired spinal blood flow and nutrition supply, accelerated spondylosis or resulted in rapid infection, and other surgical problems (259–261). While the pathophysiological mechanism and pathological characteristics of IVDD brought on by cigarette smoke remain unknown and a clear link between smoking and IVDD remains speculative, smoking appeared to increase and accelerate the chance of disc herniation through capillary constriction as an independent risk factor in patients with lumbar disc herniation (249, 257, 262–265).

Degeneration is quickened by the interaction of hereditary and environmental factors. There is currently no study that acknowledges the independent influence of environmental factors without genetic predisposition (144). However, subtypes of herniation may develop as a result of sedentary lifestyle (266, 267). The composition of the disc retains water to keep the hydrostatic pressure constant, keeping the NP elastic, flexible and able to withstand compression (268). There is convincing evidence that as people age, the likelihood of disc degeneration increases, partially as a result of the accumulation of senescent cells (269, 270). Although in mitotic arrest, these cells remain metabolically active and anaerobic metabolism contributes to increased acidity (271) and their senescence associated secretory phenotype (SASP) is likely luring more neighboring cells into the same fate (272). Lastly, obesity is linked to biomechanical alterations that lead to a variety of spinal disorders like IVDD, osteoarthritis, disc herniation, and spinal stenosis (273, 274).

## Current and future therapy options for IVDD

**Figure 3:**
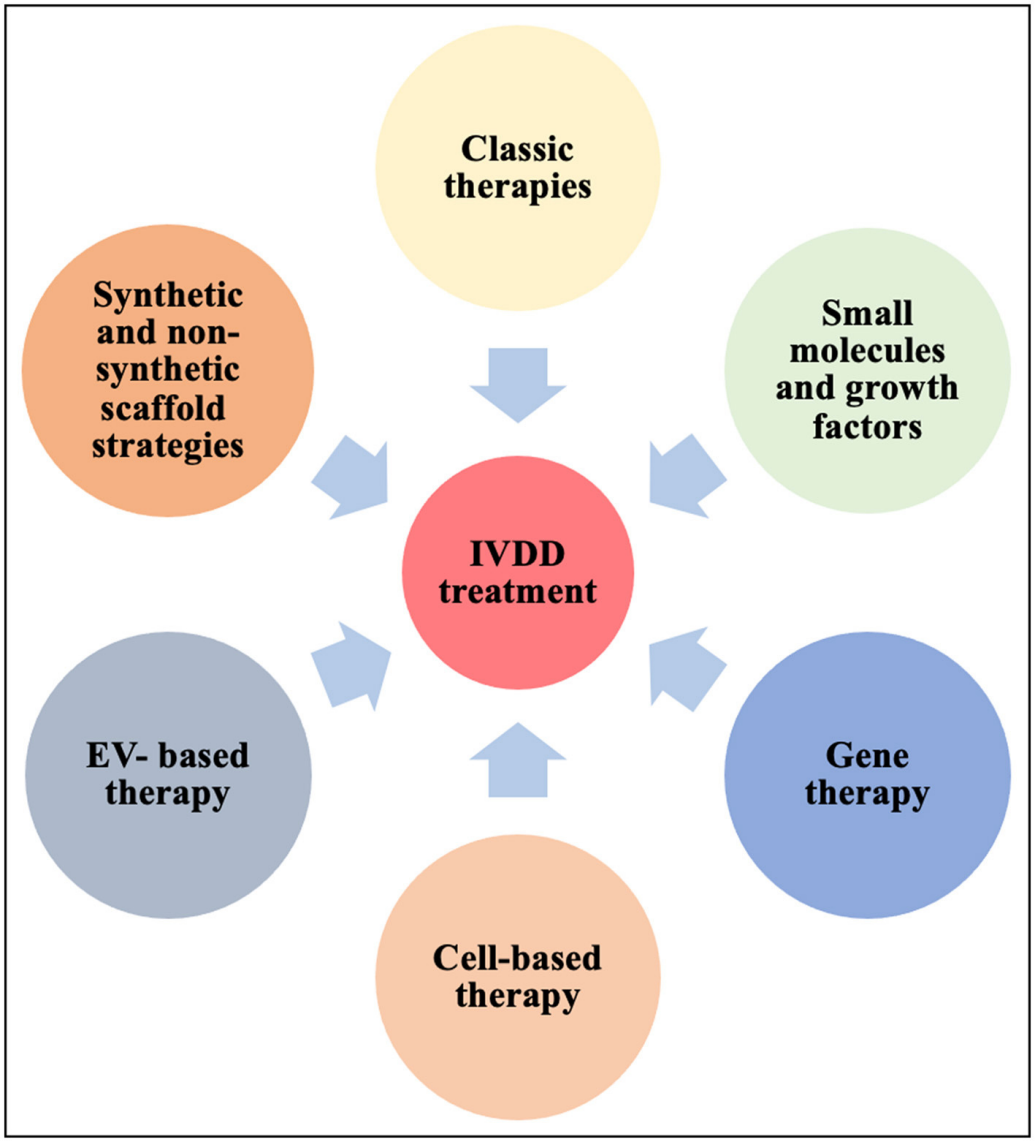
Current strategies for IVDD therapies. EV, extracellular vesicles; IVDD, intervertebral disc degeneration; MSC, mesenchymal stem cells.

IVDD is closely tied to the loss of ECM producing cells in the maturing NP. Cell survival especially in the degenerating NP environment is challenging and cell death can have complex consequences on tissue homeostasis and immunity, triggering amongst many outcomes the release of proinflammatory cytokines (275, 276). Therapeutic interventions for IVDD (Figure 3) changed substantially over the years, however no treatment leading to a cure has been established so far. Owing to the nature of the IVD, many strategies are based on endogenous approaches that aim to stimulate resident progenitor cell populations, whereas exogenous approaches try to replenish the IVD with new cells. Efforts are made to minimize cell death and SASP associated signaling cascades.

### Classic therapies

Surgery, steroids and non-steroidal anti-inflammatory drugs (NSAIDs), analgesics, opioids, muscle relaxants, and physical therapy are some of the classic therapies to alleviate IVDD symptoms like pain (277, 278) enabling short term relief, but not eradicating the problem. Radiographic imaging techniques like MRI can often demonstrate that the ongoing discomfort is caused by nerve compression (279). In recent years, regenerative medicine-based approaches along with other therapeutic interventions are gaining increased attention for advanced IVDD therapies.

### Cell therapies

Cell therapy to refurbish the IVD is an important area of research (280). As the number of healthy resident cells gradually declines during IVDD, catabolic activities take place over tissue anabolism (281). To encourage endogenous repair of the degenerated IVD, stem cells can be extracted from various sources and transplanted into ailing host tissues. Harnessing “stemness” is intriguing and includes the transplantation of transdifferentiated somatic cells, induced pluripotent stem cells and embryonic stem cells. Undifferentiated stem cells have the capacity to self-renew and multiply, giving rise to committed, differentiated cells that replenish the cell pool in a particular tissue (282). There, they may secrete growth factors and cytokines to support resident cell activity, and attract or stimulate local progenitor cells (281–283). Stem cells have been isolated from a number of tissues, including the IVD (46, 284–290). Pluripotent cells however pose a risk of tumorigenesis. Additionally, some cell types are deemed uneconomical on an individualized basis, are not fully understood in their differentiation potential, or their generation and use is of ethical concern (291–294). Among candidates that have emerged for cell-based therapies for IVDD are NC cells, chondrocytes, MSC and NP cells, some have undergone preclinical and/or clinical examinations (197). Selecting a cell type requires understanding of disc development as well as knowledge of the cellular changes induced by maturation and degeneration (6, 44, 295). Some IVD cell populations exhibit progenitor cell potential as discussed above (46, 47, 49, 50, 197, 296–299), yet would require surgery for harvesting. Autologous or allogeneic MSC gained popularity as their less-tumorigenic multipotent phenotype might be directed into the appropriate cell type via endogenous cues from the recipient tissue or ECM. MSCs, especially subcutaneous adipose MSCs, offer a promising option owing to their ease of harvest, capacity for self-renewal, multilineage potential, and immunosuppressive properties (197, 300–302). However, transplanted stem cells face delivery and survival challenges in the harsh environment of the IVD which are exacerbated in the degenerated disc (6, 205, 290, 303, 304). MPC-06-ID, a Phase 3 product candidate was developed to address IVDD related chronic pain with 6 million mesenchymal precursor cells per dose for patients who have exhausted other therapy options (www.mesoblast.com). A recent subjective review indicated that result considering impairment, pain, and quality of life were influenced by the placebo effect. Therefore, more quantifiable and objective measures such as MRI and other radiographic exams are needed (305). A study to examine the clinical applicability, safety, and efficacy of NOVOCART^®^ Disc in the repair of herniated discs requiring an elective sequestrectomy employs an autologous cell compound (306). The Sponsor has permanently halted the NOVOCART^®^ Disc development program since there was no evident advantage of the investigational intervention above standard therapies (https://www.tetec-ag.de/en.html). An updated list of clinical trials for MSC in IVDD can be seen in Table 3 (clinicaltrials.gov). Further large-scale, randomized (placebo), controlled studies for cell based IVDD therapeutics are needed.

**Table 3:** Clinical trials reported with growth factors in the context of IVDD based on data from May 2023 (www.clinicaltrials.com).

**Status**	**Type**	**Trial ID**	**Phase**	**Result**
Completed 2014	Evaluate the safety, tolerability, and preliminary effectiveness of single administration intradiscal rhGDF-5 for the therapy of early-stage lumbar IVDD (1.0 and 2.0 mg) Open label Australia	NCT01158924	1/2	Unclear if neurological, ODI and VAS outcome was an improvement, increased score for functional health and wellbeing Therapy emergent adverse effects in 14% (1.0 mg) and 4% (2.0 mg).
Completed 2013	Intradiscal rhGDF-5 (0.25/1.0 mg) Open label United States	NCT00813813	1/2	Unclear if neurological, ODI and VAS outcome was an improvement, increased score for functional health and wellbeing. Therapy emergent adverse effects in 29% (0.25 mg) and 4% (1.0 mg).
Completed 2014	Evaluate the safety, tolerability, and preliminary effectiveness of single administration intradiscal rhGDF-5 for the therapy of early-stage lumbar IVDD. (placebo/1.0 mg) Randomized, double blind study. Republic of Korea	NCT01182337	1/2	No therapy emergent adverse effects. Unclear if neurological, ODI and VAS outcome was an improvement, score for functional health and wellbeing indicates placebo effect.
Completed 2014	Multicenter, randomized, double-blind, placebo controlled, clinical trial to evaluate the Safety, Tolerability and Preliminary effectiveness of 2 doses of intradiscal rhGDF-5 (for the therapy of early-stage lumbar IVDD) (placebo/1.0 mg/2.0 mg) Randomized, double blind study. United States.	NCT01124006	2	No therapy emergent adverse effects. Unclear if neurological, ODI and VAS outcome was an improvement, score for functional health and wellbeing indicates placebo effect.
Not yet recruiting	Intradiscal and intra-articular injection of autologous platelet-rich-plasma (PRP) in patients with lumbar IVDD and facet joint syndrome. Open label.	NCT04816747	3	No results posted

ODI, Oswestry disability index (Pain Intensity, Personal Care, Lifting, Walking, Sitting, Standing, Sleeping, Sex Life, Social Life, Traveling) disability measurement scale; VAS, visual analog scale pain score.

### Extracellular vesicle therapies

**Figure 4:**
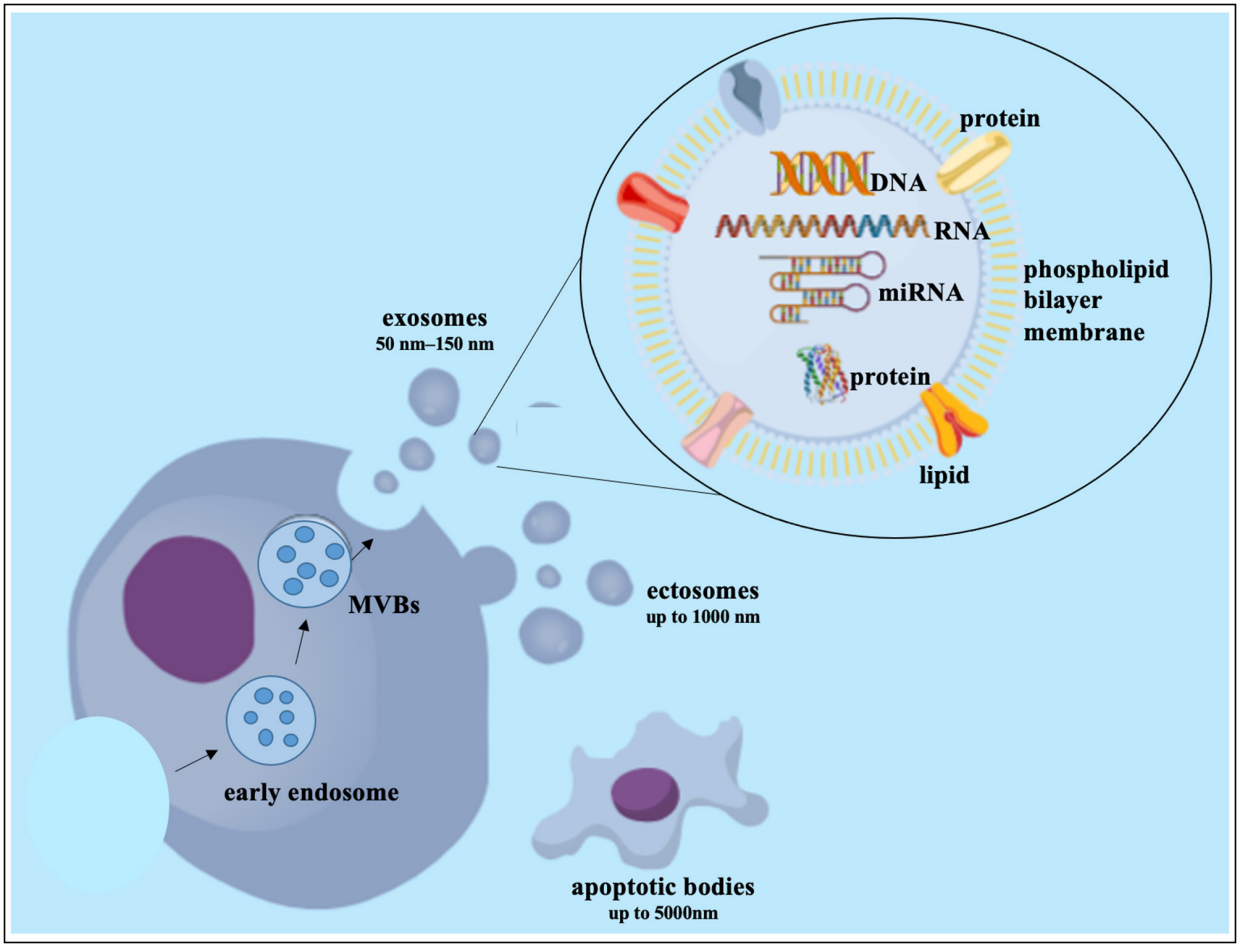
Exosome biogenesis and composition. DNA, deoxyribonucleic acid; miRNA, microRNA; MVB, multivesicular bodies; RNA, ribonucleic acid. This figure created in the Mind the Graph platform (www.mindthegraph.com).

Cell-to-cell communication is fundamental for the maintenance of microenvironment homeostasis (307). Our knowledge of cell-cell communication has improved with the development of large-scale “-omics” technologies for analyzing the secretome of cells. These technologies have also allowed us to investigate extracellular vesicles (EV) with cell-type specific cargos of proteins and nucleic acids (285, 307). Although the classification of EVs is constantly changing, they usually fall into one of three categories: Exosomes (50–150 nm) are created by the endosomal formation of multivesicular bodies (MVB). Apoptotic bodies (up to 5,000 nm) and ectosomes (up to 1,000 nm) are generated by outward budding of the plasma membrane (308, 309) as seen in Figure 4. Most cell types produce exosomes, and their release into body fluids and culture media has sparked interest in finding cancer biomarkers (310). In fact, researchers from a variety of sectors are increasingly interested in analyzing EVs produced by resident cells in the hopes of identifying specific cell or disease-related biomarkers (311). Exosomes with cell-specific proteins, lipids, and nucleic acids are now recognized as a form of intercellular communication method (312). This theory is supported by the observation that exosomes generated by parental cells may interact with target cells, causing target cell behavior and phenotypic traits to be influenced (313). Limited research has been done on IVD-derived EVs (105, 107, 311, 314). In the area of biomarkers of LBP and disc disorders, there have been a lot of encouraging research findings such as the ongoing study and validation of pertinent, correct, and sensitive biomarkers of disc disorders (315). NC-derived EVs enhanced DNA and glycosaminoglycan content in human NP cell micro-aggregates compared to untreated control conditions although the underlying mechanism and associated EV content were not examined (316). EVs derived from human NP cells of patients with lumbar degenerative disease were found to promote MSC migration and differentiation into an NP-like phenotype via the Notch1 pathway, though the precise EV content responsible for this action is unknown (88, 101). Several examples of MSC derived exosomes impact on cell survival, often through their micro RNA (miRNA) cargo affecting key signaling pathways controlling events in apoptosis and pyroptosis (276). Human UC-MSCs exosomes prevented NP cell pyroptosis by targeting METTL14 with a methyltransferase that catalyzes the m6A change (317). NP cell apoptosis decreased by miR-142-3p reducing IL1-induced inflammatory cytokine release and MAPK pathway activation (84). TNFα induced apoptosis, ECM breakdown, and fibrosis in NP cells was prevented via miR-532-5p targeting Ras association domain-containing protein 5 (RASSF5) (85). Pyroptosis in IVDD was reduced by miR-410 by binding to the pyrin domain containing 3 (NLRP3) mRNA (96, 318) and miR-26a-5p prevented pyroptosis by reducing NLRP3, IL1, and IL18 expression (317). IVDD and gait abnormalities improved through miR-4450 targeting the zinc finger protein 121 (97) and miR-141-3p via the Kelch-like ECH-associated protein 1 (Keap1)-Nuclear factor (erythroid-derived-2) like 2 (Nrf2) pathway reduced oxidative stress-induced pyroptosis in NP cells (319). In a recent systematic review it was further reported that stem cell-derived EVs can slow the progression of IVDD at the cellular, molecular and organ levels (320). Lastly, an ongoing clinical trial (NCT04849429) uses platelet-derived exosomes for IVDD and may soon provide useful *in vivo* evidence on the therapeutic effect of exosomes.

### Synthetic and non-synthetic scaffold-based therapies

Structural integrity and support can be provided via three-dimensional (3D) tissue scaffolds that enable cellular interactions between native tissues and the implant and provide structural support for the cells by mimicking cell-ECM interactions. The ECM, a composite of fibers, bioactive molecules and solutes is dynamic *in vivo* providing structure and signals to the cells that generate it (321). Regenerative medicine makes use of “inductive” properties of the ECM. In the early 1960s scaffolds were created as cell substrates to resemble the niche in which cells thrive, enabling cells to attach, differentiate and proliferate (17, 322). Ideally such a scaffold would be non-cytotoxic, biocompatible and eventually biodegradable (323). Recent research demonstrates promising biomaterials and processing techniques for IVD repair or regenerative strategies. Composite scaffolds that allow for simultaneous regeneration of cells and ECM would be most beneficial because IVDD affects both (324). In the past, 3D biomimetic scaffolds were created using a variety of methods: solvent casting, freeze drying, phase separation, leaching and electrospinning (325–327). Bioprinting is also investigated for the IVD. Although in its early stages, using this cutting-edge method could enhance the creation of IVD-based scaffolds (328). Based on the polymer used, these scaffolds can be considered synthetic or non-synthetic (natural).

#### Natural materials

The most prevalent protein in mammals, collagen, is employed extensively in biomedical procedures and its function is likely crucial for IVD regeneration (289, 329). Owing to its minimal antigenicity, atelocollagen is recognized as one of the best basic matrices for implantable materials (330). A mechanically stable, manageable, honeycomb-shaped atelocollagen scaffold promotes the development of high-density cell cultures (330) and may be beneficial as a 3D scaffold in tissue engineering given these properties (331). Silk scaffolds provide strength and stability through compressive and tensile properties. Silk fibroin proteins are synthesized by silkworms and other insects and are biodegradable (332, 333). Once implanted, the silk scaffold would decay slowly enough to permit healthy tissue growth (332, 334). Silk scaffolds are of interest for AF tissue engineering owing to their mechanical properties (335). The naturally occurring polymer chitosan is a very adaptable biomaterial (336). It comes from a natural and regenerative source: crab shells (337–340). Chitosan possesses a hydrophilic surface that encourages cell attachment and growth, and its degradation products are non-toxic (341). Alginate is utilized in a number of biomedical applications, including tissue engineering and drug delivery, because of its qualities in terms of biocompatibility, biodegradability, non-antigenicity, and chelating ability (342–346). A hybrid alginate/chitosan scaffold promotes ECM deposition, enhances AF cell proliferation, and degrades more slowly than a pure alginate scaffold (346). Another biomaterial which develops a stable hydrogel structure as a result of gelation is gellan gum (347). A 3D gel network that can be employed as a matrix for cell seeding is created when untangled sections of polysaccharide chains connect to orientated bundles of double helix structures (348–350). Natural polymers support cell adhesion and function (351).

Decellularized ECM-based scaffolds have drawn a lot of interest and have begun to be utilized extensively in a variety of tissues (heart valves, vascular grafts, cornea, etc.) (352–356). However, decellularization protocols cannot rely on perfusion in the largely avascular IVD and a balance between complete cell removal to avoid inflammatory triggers and ECM preservation to allow for bioactivity is important (357, 358). Decellularized scaffolds are currently commercialized for numerous therapeutic uses because of their pro-regenerative capabilities, and they may offer a promising alternative for IVD regeneration (357, 359).

#### Synthetic materials

Necessary forms and implants can also be created from synthetic polymers. Synthetic biodegradable polymers can generate stable porous materials that are predesigned 3D scaffolds and do not melt or disintegrate in *in vitro* tissue culture settings (360). The synthetic biodegradable polymers most frequently employed in tissue regeneration are aliphatic polyesters (351). The ester groups in these polymers' backbones are often hydrolyzed to produce deterioration, which can be regulated depending on the polymer's composition, structure, and molecular weight (361). A ring-opening polymerization of the monomers (lactide and/or glycolide) is a typical method for producing polylactide (PLA), polyglycolide (PGA), and their copolymer poly (lactide-co-glycolide) (PLGA) (362). These polymers are among the few synthetic polymers that the U.S. Food and Drug Administration (FDA) has approved for human clinical applications, such as surgical sutures and some implanted devices. Synthetic polyesters with a wide range of applications in AF tissue regeneration include poly ε-caprolactone (PCL), PGA, PLA and copolymers produced from these monomers (363, 364). PCL has been widely employed as a biocompatible polymer with reasonable cost and high mechanical qualities for electrospun fibrous scaffolds (365). Many synthetic polymers are hydrophobic with restricted water absorption requiring modifications for cell attachment. Some limitations in their biomedical applications, may be solved by including other polymers, such as natural or synthetic proteins and polysaccharides (366). A development in the field of synthetic scaffolds is the use of “conductive” or “smart” biomaterials. Conductive materials are typically polymer or nanomaterial-based additives to the scaffold allowing for the transfer of electromechanical signals to target cells (367). A conductive effect could also be achieved with natural polymers like collagen based on a described piezoelectric effect under load for ordered collagen fibers, especially collagen I (368). A piezoelectric potential of the AF and to a lesser degree NP tissue was described. This approach could facilitate more effective mechanically induced tissue remodeling and cell homing in the IVD (369).

NuCore^®^ injectable nucleus hydrogel (Spine Wave, Inc., Shelton, CT, USA) as a substitute for NP tissue lost to herniation and microdiscectomy was investigated and seemed to prevent the disc from collapsing too soon after microdiscectomy (370). The FDA approved Discseel^®^ which relieves chronic neck and low back discogenic pain offers a procedure to effectively repair discs to their normal states, both mechanically and biochemically (371). Owing to the relative short timeframe of follow up with some of these procedures not much clinical evidence to support these therapies is available. Also, secondary effects of IVDD, such as spinal stenosis and muscle fatigue caused by lumbar lordosis and loss of sagittal stability of the spine, may be more excruciating than structural transformation in the disc themselves (372).

### Small molecules and growth factors-based therapies

Small molecules are substances that attach to certain biological molecules and aid in the regulation of a specific biological process (222). Small molecules can be taken orally (373). The maximum molecular weight for a molecule that needs to quickly diffuse through the cell membrane and be absorbed by the digestive system is 900 Daltons (374). Small molecules can significantly alter signaling transduction and gene transcription by intervening on specific signaling pathways regulating cell physiology and function (375). There are various benefits of using small molecules as a therapeutic agent. They cause fewer immune response in the host owing to their small size, and are considered to have anti-inflammatory, anti-apoptotic, and anti-oxidative effects accompanied by anabolism and anti-catabolic effects (222). The anti-inflammatory effect of small molecules such as berberine, morin, notoginsenoside R1, cannabidiol, curcumin, icariin, resveratrol, epigallocatechin gallate, naringenin, and tofacitinib was shown by the downregulation of IL1 and TNFα levels in IVD cells in a number of *in vitro* studies (222, 375, 376). Src homology region 2-containing protein tyrosine phosphatase 2 (SHP2) is an important contributor to the development of IVDD, and its small molecule inhibitor SHP099 prevented SHP2 expression and NP cell degeneration (377). Following toll-like receptor (TLR) 2/6 agonist induction, o-vanillin reduced TLR2 expression and SASP (378). Other small molecules acted in a multipotent manner. Curcumin showed cell-type and experiment dependent pro-apoptotic or anti-apoptotic effects. In IVDD it reduced the activity of proinflammatory cytokines by inhibition of the nuclear factor kappa B (NF-kB) and mitogen-activated protein kinase (MAPK) pathways, protected mitochondria and induced autophagy via its reactive oxygen species (ROS) scavenging capacity (379–384). Icariin, a bioactive and peroxylated flavonol glycoside compound isolated from *herba epimedii* or horny goat weed was investigated as a therapy of articular cartilage degenerative diseases (385). Its anti-oxidative and mitochondrial protective effects were attributed to the activation of the PI3K/Akt and Nrf2 signaling pathways, culminating in decreased ROS production and programmed cell death in NP cell (385, 386). Melatonin induced parkin-dependent mitophagy, also protected mitochondria (380, 387) and exhibited anti-inflammatory effects by inhibiting IL1 release and NLRP3 primed pyroptosis (388). When high hyperglycemia caused mitochondrial damage in end plate cells, alpha lipoic acid prevented apoptosis by increasing mitochondrial membrane potential (389).

Growth factor (GF) therapy involves the injection of bioactive molecules into the IVD to promote ECM production, prevent degeneration, and decrease inflammation (390, 391). GFs are peptides that bind to receptors and trigger physiological processes such as protein synthesis, differentiation, apoptosis, and cellular proliferation (392). Bone morphogenic proteins (BMPs) and other transforming growth factor (TGFβ) members, which promote osteogenesis and chondrogenesis, are the most well-known GFs in spine and orthopedic therapies (215). In an IVDD mouse model, TGFβ inhibitors decreased Nerve growth factor (Ngf) expression, indicating that TGFβ may control Ngf expression *in vivo* (393). Other GFs like BMPs, platelet derived growth factors (PDGF) and epidermal growth factor (EGF) inhibit proinflammatory cytokines including IL1, IL6, TNFα, MMPs, nitric oxide, and prostaglandin E2 (PGE2) and decrease catabolic activity (391, 394). The biological half-life of GFs is only a few hours to days, making it unsuitable for restoring degenerative discs when GF stability or long lasting effects are required (391, 394). Platelet-rich plasma (PRP) contains a variety of GFs (395, 396). PDGF decreased the percentage of apoptotic AF cells *in vitro* after of serum deprivation (397). An updated list of clinical trials for GFs in IVDD can be seen in Table 4 (clinicaltrials.gov).

**Table 4:** Clinical trials reported with mesenchymal stem cells (MSC) in the context of intervertebral disc degeneration (IVDD) based on data from May 2023 (www.clinicaltrials.com).

**Status**	**Type**	**Trial ID**	**Phase**	**Result**
Completed 2017	IVDD therapy with allogeneic MSC. Randomized, triple blind study. Spain	NCT01860417	1/2	MSC vs. Mepivacaine not yet available in database. Reproducible cell expansion and satisfactory quality control tests (398)
Completed 2017	Use of autologous BM-MSC in patients with lumbar IVDD. Open label. Spain	NCT01513694	1/2	No results posted
Completed 2015	Safety and preliminary efficacy of mesenchymal precursor cells in subjects with lumbar back pain. Randomized, double blind. United States, Australia	NCT01290367	2	No results posted
Completed 2013	Study of 3 doses of NeoFuse combined with MasterGraft granules in subjects requiring posterolateral lumbar fusion. Randomized, open label. United States	NCT00549913	1/2	No results posted
Withdrawn 2015	Autologous AD-MSC transplantation in patient with lumbar IVDD. Open label. Republic of Korea.	NCT01643681	n/a	No results posted. Unwilling to continue clinical trials.
Withdrawn 2012	Lumbar IVDD therapy with autologous BM-MSC. Open label. No location data.	NCT02440074	1/2	No results posted. Not funded. Administrative formalities.
Withdrawn 2022	MSC for lumbar IVDD. Randomized, open label. Unites States	NCT03692221	1	No results posted. Stalled due to COVID-19
Withdrawn 2011	Safety and efficacy of NeoFuse in subjects requiring posterolateral lumbar fusion. Open label. United States	NCT00810212	1/2	No results posted. Withdrawn for better study design (Mesoblast).

AD-MSC, adipose-derived mesenchymal stromal cell; BM-MSC, bone marrow derived mesenchymal stem cell.

### Gene therapy

The use of nucleic acids such as DNA or RNA to cure a disease is known as gene therapy (399), often targeting monogenic congenital diseases or cancer. A plasmid (400) or oligonucleotide can be used (401). Gene therapy's potential long-term efficacy is a key benefit (402). Transfected cells that have received a therapeutic gene produce the desired gene products (RNAs or proteins). Stable transfection facilitates long-term expression of a transgene even in dividing cells if the foreign gene integrates into the host genome, however this can come at the expense of insertional mutagenesis. Cells that have been transiently transfected with an episomal vector also express a foreign gene but the foreign gene will be lost in dividing cells (403). IVDD is a chronic problem (129, 402, 404–406). Retrovirus (RV), lentivirus (LV), adenovirus (AV), and adeno-associated viruses (AAV) are common vectors (407). Replication incompetent RV were used *in vitro* to deliver DNA to cells purified from bovine coccygeal vertebral endplates suggesting that local gene therapy may be used to treat disc degeneration (129, 408, 409). Non-human LVs are considered apathogenic in humans but can transduce human cells. Replication-incompetent LV vectors are available (410). To demonstrate that LV-mediated MMP3 knockdown may lessen IVDD, LV-MMP3-shRNA and/or LVSox9 were administered to rabbit lumbar discs. This significantly delayed the progression of IVDD and increased collagen type II and proteoglycan expression (128). Insertional mutagenesis remains a concern associated with highly efficient RV and LV vectors (411). The AV genome persists in an extrachromosomal state. Standard recombinant AV vectors can carry up to 7.5 kb of foreign DNA (412). To further increase the packaging capacity to more than 30 kb AV genes are provided in-trans by a helper virus (413). A recombinant AV vector was used to deliver the *lacZ* gene to female New Zealand white rabbit NP cells *in vitro* and *in vivo* (130). The AV-*lacZ* construct was directly injected into the NP of the rabbit's lumbar IVD for the *in vivo* model. The successful transduction of disc NP cells was demonstrated by X-Gal (5-bromo-4-chloro-3-indolyl-D-galactopyranoside) staining and reporter gene expression persisted *in vivo* for at least 12 weeks. This study demonstrated the promise of direct gene therapy for a treatment of IVDD by successfully delivering a foreign gene to the IVD (414, 415). AV have several different serotypes, including 51 in humans (412); Ad5 is the most common and 45–80% of the population has neutralizing antibodies against this serotype (412). Unfortunately AV vectors in general can cause severe and even lethal inflammatory reactions (416, 417). AAV's are used more recently as non-pathogenic, generally non-integrating gene therapy vectors suitable for dividing and non-dividing cells. However, it is challenging to generate the high titers needed for human clinical studies and the packaging capacity is limited (415, 418). The activatorprotein-2 (Ap2α) impacts IVDD via controlling the expression of Tgfβ and Smad3 (132). Rat IVDs injected AAV-Ap2α and AAV-Tgfβ, increased the expression of Acan, Collagen II and decreased the expression of Mmp2, Mmp9, and Smad3 in NP tissue (132). However, in general, viral vector based gene therapy carries a risk of viral component-related complications (419, 420).

The post-transcriptional RNA interference (RNAi) mechanism evolved as a crucial biological strategy for targeted gene silencing (402). The reporter genes firefly and renilla luciferase were downregulated in NP cells *in vitro* in a co-transfection experiment and achieved considerable inhibition of reporter gene expression in both cell types for 3 weeks, suggesting siRNA-mediated gene silencing as effective in NP cells (421). Moreover, in rat coccygeal IVDs, siRNA-mediated RNAi remained active for at least 24 weeks to down regulate *in vivo* expression of the endogenous Fas ligand, as well as a reporter gene (422). MRI and histological studies showed that a single injection of ADAMTS5 siRNA prevented NP tissue breakdown after annular puncture *in vivo* (423). Apoptosis in the discs was also significantly reduced by siRNA therapy intervention against Caspase3 and ADAMTS5 (424). Inhibiting *TLR4* and overexpressing *Klotho* via RNAi in a rat IVDD model decreased ROS induced inflammation (133). Klotho promotes antiaging through the modulation of numerous signaling pathways, including TLR4/NF-kB signaling (133, 425). However, *in vivo* applications could be hampered by RNAi associated immune stimulation, off-target effects and the low number of target cells in the IVD (426). There were no clinical trials reported for gene therapy-based therapeutics in IVDD until now (clinicaltrials.gov).

Most recently CRISPR/Cas9 (427) was also added to the growing toolkit for IVDD therapy development. Potential applications for CRISPR/Cas9 gene editing, targeting or labeling to enhance IVD research by generating new disease models, new means of studying IVD cell phenotypes and possible clinical translations thereof were suggested and reviewed (428). As a promising recent example, AAV delivered CRISPR/Cas9 to target β-catenin reduced IVDD in the mouse model (429) and CRISPR epigenome editing systems could be introduced into pathological human IVDs *in vitro* using LV vectors to control expression of inflammatory receptors. This could suppress negative impacts of inflammatory cytokines in the IVD. TNFR1 epigenome-edited cells showed decreased NF-kB activation, reduced apoptosis, and suppression of catabolic gene expression changes (430).

## Discussion

The IVD at first glance appears as a simple organ comprised of just two major tissue types with few residing cells under extreme mechanical or physiological stress yet it is possibly one of the most challenging enigmas in the vertebrate body to solve. Therefore, despite IVDD being a primary health concern, it still must find a permanent cure. IVDD decreases the quality of life by causing chronic discomfort and discogenic pain due to multifactorial changes in the degenerating IVD as previously described in detail (431). The IVD is susceptible to a variety of risk factors and can deteriorate because of a pathologic cascade resulting in metabolic and cellular changes in IVD cells. Classic IVDD therapies were reviewed before (431) and are available in health centers, but often encounter a “roadblock” in that they only relieve symptoms but do not restore structure and functionality to the disc. Surgical options for IVDD are often ambiguous and carry underlying hazards and complications, hence, they should only be used after conservative measures have failed, as their outcome depends on a surgeons' experience and technical expertise, as well as a patients' comorbidities (391).

**Figure 5:**
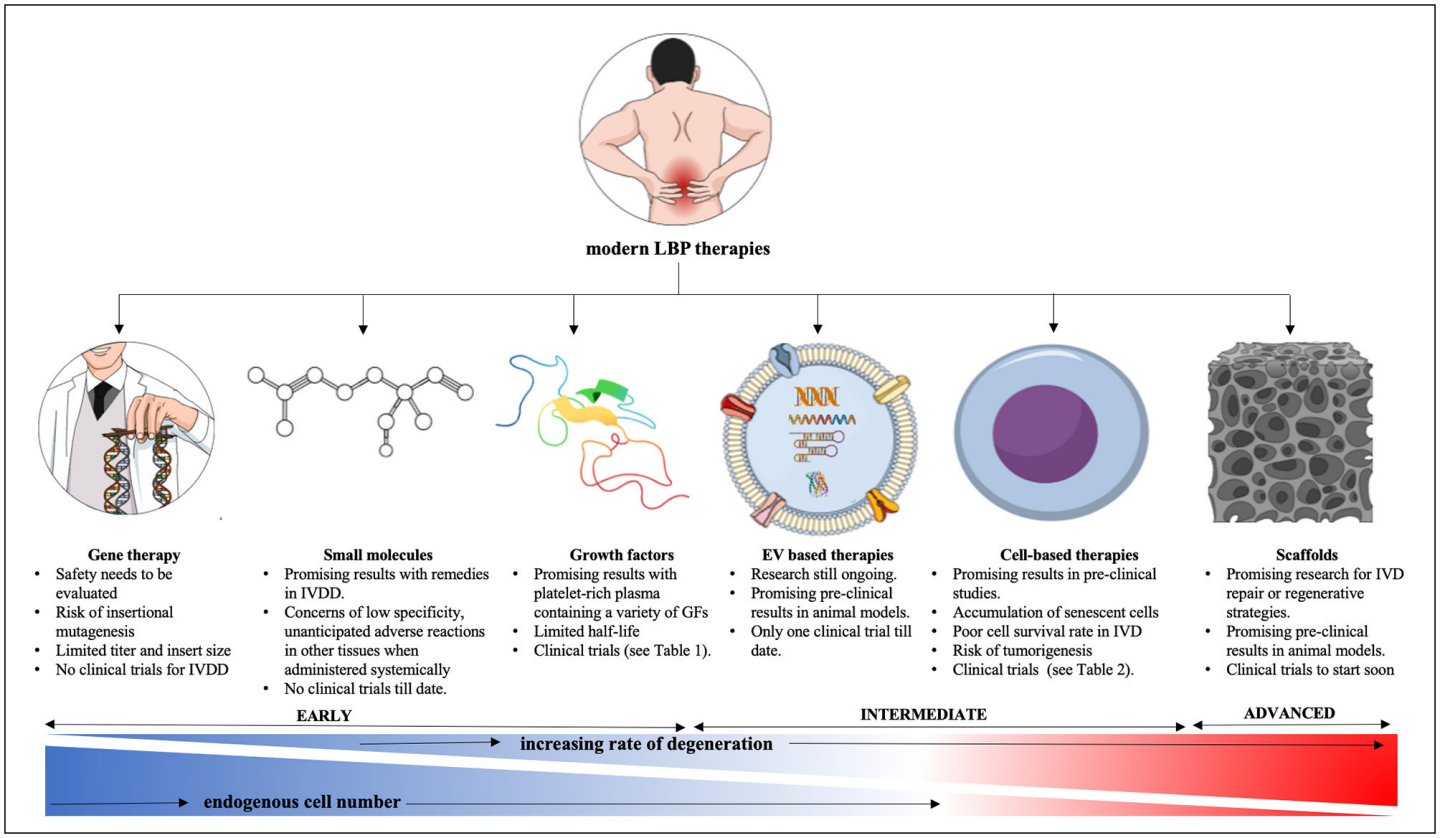
Summary of modern therapy approaches for lower back pain from IVDD. IVD, intervertebral disc; IVDD, intervertebral disc degeneration; GF, growth factor; AAV, adeno-associated virus. This figure was created in the Mind the Graph platform (www.mindthegraph.com).

Advanced therapies of low back pain as summarized in Figure 5 show some promising results in mostly animal studies (Table 1) but still have their own safety concerns and limitations. To start, these novel interventions once intended for clinical applications first require the approval by appropriate government bodies such as the FDA in the United States, European Medicines Agency (EMA) in Europe or the Central Drugs Standard Control Organization (CDSCO) in India. Based on successful outcomes of pre-clinical studies an investigational new drug application (IND) can be filed with the FDA triggering several phases and years of clinic trials with uncertain outcome for the investigator and high financial risk for sponsors as previously described (320). Despite all, progress is evident and current, and future research will hopefully translate many of these cutting-edge technologies from benchside to bedside as alternative IVDD therapies despite plenty of challenges that remain to be addressed.

Owing to the avascular nature of the IVD, systemically applied therapies are less suitable. Therefore, exogeneous and endogenous interventions would require intradiscal injections of cells, hydrogels, GF, small molecules, viral vectors or combinations thereof. Such injections produce a bolus of compressed fluid at the injection site that may take a long time to diffuse into the surrounding tissue due to a high degree of stiffness and limited permeability in NP tissue. This pressure may cause fluid to leak through the AF defect after the needle retraction (432–434). The severity of AF disruption can vary depending on the needle gauge used, stiffness and fluid viscosity and would require oversight to assess the danger of leakage, especially for more advanced therapies such as gene therapy involving viral vectors (435–438). While the avascular nature of the NP limits the use of systemic interventions it could keep side effects limited after intradiscal delivery, provided no leakage at the injection site. Despite promising potential, currently, small molecule drugs have little clinical relevance in IVDD as they do not appear to offer a significant advantage over NSAIDs (375, 439, 440). The reasons might be low specificity, the avascular nature of the IVD and unanticipated adverse reactions in other tissues when administered systemically. To date, most *in vivo* studies have focused on rodent models, and more appropriate translational models are needed for an honest assessment of safety and efficacy of small molecules as alternative strategy to NSAIDs.

Recent advancements in sequencing technologies identified genetic defects associated with IVDD and LBP and will enable more personalized therapy approaches. At the same time, increased knowledge of cellular events at the molecular level facilitates more targeted therapies with recombinant or xenofree bioactive molecules or inhibitors thereof down to modulations of intracellular signaling pathways, for example those involving cytokine triggered inflammation, regulated cell death or SASP in the IVD (222, 441, 442). However, despite success in animal models, IVDD human gene therapy in the classic sense of gene delivery is unlikely to be a mainstream intervention any time in the near future as IVDD is not a monogenic disease and current technologies do not allow to effectively and safely alter multiple genes *in vivo*. Safer viral and non-viral vectors with improved cargo capacity and better transfection efficiency at a lower dosage alongside reduced immune response activation are needed for increased safety and efficacy of gene therapy in general. Promising work using engineered AAVs and serotypes with different tropism were underway for several diseases until recent setbacks sent once again alarming signals through the gene therapy community (443). Gene therapy for IVDD ideally employs vectors that can target NP cells specifically through unique cell surface/viral capsid protein interactions, however, this could arise as one of the bigger challenges given that the adult NP cell population is heterogeneous and suitable NP cell unique cell surface markers have yet to be discovered (41, 42). RNAi as tool to downregulate proinflammatory responses seems more promising. Recent research showed that miR-370-3p-regulated circular RNA (circRNA) *PKNOX1* controlled the expression of KIAA0355, which impacted on IVDD progression, hence *circPKNOX1*-based therapy may become useful (444). However, *in vivo* off-target effects remain a concern for RNAi and CRISPR gene editing for now. A better understanding of the pleiotropic impact of bioactive molecules like miRNAs on various, often connected signaling pathways including those critical in inflammatory response, senescence, cell cycle arrest and regulated cell death is crucial for safety and efficacy (222).

Endogenous cell-based therapies stimulating native IVD progenitor cells depend largely on effective and safe delivery of the stimulant, while exogenous approaches transplanting autologous/allogenic cells depend on the ability of those cells to settle, survive and be productive in a challenging or degenerated environment. Cell therapies struggle with the accumulation of senescent cells, a poor survival rate of transplanted cells and the necessity of correct differentiation (445). Increased cell death post transplantation could trigger inflammasome related pyroptosis and further aggravate IVDD. Many *in vitro* studies have investigated the efficacy of MSCs in preserving and reactivating NP cells isolated from healthy or degenerate discs by maintaining or enhancing ECM synthesis as well as by encouraging upregulation of NP markers, which are diminished within the diseased disc (197, 446–448). In a variety of studies, MSCs such as those produced from bone marrow (BM-MSCs), adipose tissue (AD-SCs), and umbilical cord (UC-MSCs) were employed alone or in combination with biomaterial scaffolds and carriers to repair and regenerate the ailing IVD (137, 449, 450). However, if non-autologous cells are used the problem of host rejection presents itself and even if the cells are tolerated, it remains unclear if these added cells can survive long enough under conditions they encounter in the degenerate IVD (285). Few studies have examined how transplanted cells interact with the native disc microniche. However, some evidence backs the delivered cells' ability to reduce inflammation in degenerating discs (6, 451). *In vitro* data from 2D culture where required culture supplements such as serum or glucose and frequent medium changes might not reflect a natural IVD environment need to be evaluated with skepticism. Further large animal and advanced organ culture models, as well as clinical trials, are needed to confirm findings from these *in vitro* experiments. Numerous animal models were used in preclinical research examining cell therapies for IVD regeneration (452). Mechanical, enzymatic, or surgical methods can be used to study disc degeneration in a variety of species, including mice, rats, rabbits, pigs, sheep, goats, cows, and dogs (Table 1). Yet comparative interpretations are challenging and frequently do not yield knowledge that is easily applicable to human studies owing to a lack of agreement between different animal models (453). In particular distinctions in NP cell composition, the variable persistence of NC cells, as well as biomechanical differences hamper the translatability of small animal models (37, 41, 46, 165, 223, 454–459).

The recently developing field of EV based IVDD therapies faces challenges and bottlenecks with production cost, quality assurance of batch-to-batch homogeneity, and long-term stability of EVs. High purity production of EVs is often based on costly differential ultracentrifugation or affinity chromatography (460). The International Society for Extracellular Vesicles (ISEV) so far proposes only minimal guidelines for EV isolation and functional analysis and a range of investigator determined EV isolation and characterization methods exists (320, 461, 462). Cold chain storage for EVs was suggested but different opinions on how storage affects EV quality exist as well (463–465). A range of responses in EV recipient cells or EV parent culture conditions as well as different interaction modes between cells and EV types might complicate the interpretation of regenerative outcomes (320). Despite success in the purification of exosomes, the exact molecular mechanisms of exosome function are still under investigation. Establishing large-scale upstream and downstream manufacturing processes, accurate dosing regiments and efficacy evaluations will likely present major obstacles for quality EV-based therapeutics, yet it will be important to safely implement their application for IVDD therapy (445).

Progress made in tissue engineering over the years using a combination of natural and synthetic biomedical scaffolds, cells and bioactive molecules represents an exciting new era. In clinical trials these approaches often fail to address discogenic pain (372). For example, to date no research on ectopic sensory nerve distribution after MSC delivery to the painful disc is available. Detailed reviews and discussions of different scaffold types exist, and a “holistic” approach for IVD regeneration was emphasized by simultaneous NP, AF and CEP repair (368). Successful strategies to replace IVD tissue with non-biological scaffolds must address the unique biological shock absorbing function of the NP and/or the ECM-provided structural architecture such as the angle-ply arrangement in the AF or the spacing of different size fibers in the NP alongside the importance of continued CEP porosity, as a whole facilitating inductive and permissive signals for cells and tissue homeostasis. In light of the abundance of studies aimed at IVD regeneration presented in the literature, regeneration of CEPs is rarely addressed, despite being a significant source of nutrients and water supply for the IVD (368). A recent study found that the human CEPs have a distinct structure and, ECM composition when compared to the NP, AF, and articular cartilage (466), while others investigate CEP composition for diagnostic purposes (467) or how impaired CEP healing after surgery relates to IVDD (468). Generally, research on CEPs and the AF still does not have the momentum seen in NP research, therefore a need to include AF and CEPs more in overarching regenerative research and the development of therapeutic strategies remains.

In summary, promising IVDD therapies are developing in different areas, and possibly the combined effort will lead to biocompatible scaffolds loaded with protected bioactive molecules, EVs and/or MSC that can mobilize and recruit local progenitor cells. Examples of such efforts are underway. In a preclinical IVDD rabbit model, platelet-derived growth factor BB (PDFG-BB) delivery in a thiol-modified hyaluronic acid hydrogel significantly reduced disc degeneration by preventing apoptosis and raising collagen-3 production, preserved disc structure, and enabled biomechanical functions (70, 200). Combining a thermosensitive acellular ECM hydrogel with AD-MSC-exosomes to create an injectable functionalized ECM hydrogel could prevent pyroptosis in rat discs by lowering the expression of NLRP3 inflammasomes and minimizing the inflammatory response (469). However, additional data from pre-clinical research, clinical trials and long-term follow up assessments will be needed to ensure safety and efficacy of any approach. Several recent scRNASeq and GWAS projects provide very valuable data to better understand IVDD and it would be constructive to the field to expand these studies to more age groups, all genders, ethnicities, and stages of IVDD to identify master regulators in NP development and IVDD progression. IVDD is multifactorial and likely results from a combination of environmental risk factors and genetic predisposition. An overarching concept of modern therapies for IVD tissue homeostasis relies on the introduction, maintenance or stimulation and directed differentiation of stem/progenitor cells supported by suitable scaffolds preventing triggers of senescence or regulated cell death.

## Author contributions

AS, TL, and PK contributed to drafting this manuscript. All authors contributed to the article and approved the submitted version.
